# Revolutionizing PIP joint fracture treatment: A case of surgical precision and rapid recovery

**DOI:** 10.1016/j.ijscr.2024.109813

**Published:** 2024-05-28

**Authors:** Filippo Pantaleoni, Paolo Boccolari, Roberto Tedeschi, Danilo Donati

**Affiliations:** a Department of Biomedical and Neuromotor Sciences, Alma Mater Studiorum, University of Bologna, Bologna, Italy; b University of Modena and Reggio Emilia, Largo del Pozzo 71, 41124 Modena, Italy; c Physical Therapy and Rehabilitation Unit, Policlinico di Modena, 41125 Modena, Italy; d Department of Hand surgery and Microsurgery, University Hospital of Modena, Modena, Italy; e Clinical and Experimental Medicine PhD Program, University of Modena and Reggio Emilia, Modena, Italy

**Keywords:** Case report, Proximal interphalangeal joint (PIPJ) fracture-dislocation, WALANT technique, Surgical innovation, Joint stability

## Abstract

**Introduction:**

Proximal interphalangeal joint (PIPj) fractures are a common yet challenging injury, particularly in athletes. This case study explores innovative surgical techniques combined with targeted rehabilitation to optimize recovery and functionality.

**Case presentation:**

A 20-year-old male soccer goalkeeper sustained a severe Proximal Interphalangeal Joint fracture-dislocation of the third finger during a game. He was treated using the wide awake local anesthesia no tourniquet (WALANT) technique and a Medartis TriLock plate, originally designed for the proximal phalanx but adapted for use on the middle phalanx.

**Clinical discussion:**

Immediate postoperative mobilization was facilitated by the WALANT technique, enhancing pain management and functional recovery. The adaptation of the TriLock plate, typically not used in this context, proved crucial for stabilizing the complex fracture. Follow-up included regular physiotherapy, focusing on mobility exercises and strength training, which were instrumental in the patient's quick return to sport.

**Conclusions:**

This case underscores the effectiveness of combining innovative surgical adaptations with early rehabilitation in treating complex hand injuries. Such approaches can lead to successful outcomes, significantly improving recovery times and functional results in athletic populations. This strategy may set a precedent for future treatment protocols in sports-related hand injuries.

## Highlights

• Innovative joint surgery method
• Custom plate for joint repair
• Immediate movement post-surgery
• Complete finger motion regained
• Athlete's hand fully rehabilitated

## 1 Introduction

Proximal interphalangeal joint (PIPJ) fractures are common and challenging injuries, especially in athletes. These fractures often result from trauma, such as hyperextension or direct impact, leading to joint instability and impaired function. Traditional treatment methods, while effective in some cases, often struggle with issues like prolonged recovery times, joint stiffness, and incomplete functional restoration. These problems underscore the need for innovative surgical techniques to enhance outcomes. This case report proposes a novel technique utilizing the Medartis TriLock plate adapted for the middle phalanx, combined with the WALANT (wide awake local anesthesia no tourniquet) approach, to address these challenges and improve patient recovery and joint function [[1], [2], [3]]. This joint is vital in gripping and is a major contributor to the overall digital function of the hand [4,5]. It is frequently subjected to injuries during sports activities that involve handling or interacting with a ball, exposing it to various forms of traumatic impacts.

Among the more mild forms of injuries at this joint are avulsions, which typically occur due to a hyperextension motion of the PIPj. This type of injury can lead to the detachment of the volar plate (VP) or the avulsion of a bone fragment from the base of the middle phalanx (P2). Despite the potential severity of these injuries, an isolated volar plate injury is usually not sufficient to cause a dorsal dislocation of the P2, thanks to the structural support provided by the proper and accessory collateral ligaments that maintain joint integrity [6,7]. However, when the trauma is more significant, there is not only a risk of avulsion of the volar plate but also the possibility of rupture or disinsertion of the collateral ligaments. This more severe condition can lead to the dorsal dislocation of P2. The most extreme case of trauma, known as impact fracture, typically occurs when a longitudinal force impacts the fingertip while the joint is flexed [6,8,9]. This force drives the base of the middle phalanx (P2) into the head of the proximal phalanx (P1), causing an articular fracture at the base of P2 [7]. The stability of the PIPj and its dorsal fragment is crucially dependent on the size of the volar fragment. A larger volar fragment, exceeding 50 % of the joint surface, will almost certainly lead to the dislocation of the dorsal fragment. This is due to the mechanical forces exerted by the central slip and the superficial flexor tendon, which remain attached solely to the volar fragment. The integrity of the volar plate and collateral ligaments are thus critical to maintaining joint stability in such cases [10,11]. The treatment strategies for fracture-dislocations of the base of P2 are guided by the extent of the joint surface involvement. For lesions affecting less than 30 % of the joint surface, conservative treatment is generally sufficient. For those involving 30 % to 50 % of the joint surface, the choice between conservative and surgical intervention depends on the specific stability of the PIPj. Lesions where the involved surface is 50 % or greater invariably require surgical intervention to restore joint function and stability [12,13]. This decision-making process is crucial for ensuring optimal recovery and functionality of the joint post-injury [9,[14], [15], [16], [17], [18]].

## 2 Patient information

**Fig. 1:**
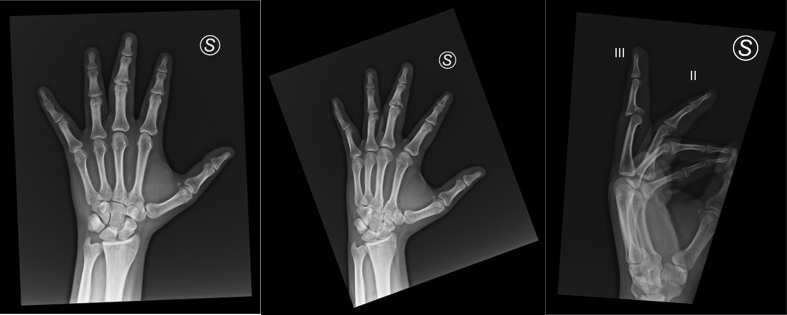
X-ray visualization of PIP joint fracture-dislocation. Lateral X-ray of the left hand displaying a significant fracture-dislocation at the PIP joint of the third digit, labeled as ‘III.’ Notable is the dislocation's alignment compared to the unaffected second digit, labeled as ‘II.’

The patient is a 20-year-old white European male and a right-handed soccer goalkeeper. He suffered a traumatic injury during a soccer match on October 14, 2023, after a forceful collision with the ball while making a save. The exact mechanism of the injury is unclear but likely involved axial trauma. He immediately sought care at a nearby emergency room, where X-rays (Fig. 1) and a medical examination confirmed a fracture-dislocation of the PIPj of the third finger on his left hand. Initially treated with a plaster splint for immobilization, he was subsequently referred to the hand surgery department. There is no reported relevant past medical or surgical history, drug history, or family history. Social history details, including smoking status or accommodation type, are not provided.

Upon assessment in the hand surgery center, the volar fragment was found to comprise about 50 % of the joint surface, corresponding to a Grade 3 injury according to the Hasting and Carroll classification [19], with dorsal dislocation and instability of P2 upon manual reduction. Notable were a complete lack of movement and severe pain. The clinical decision was made for surgical intervention using the WALANT technique (wide awake local anesthesia no tourniquet) with a Bruner palmar approach to the PIPj. During the surgery, a Medartis TriLock plate (Medartis AG, Switzerland) was used to act as a buttress plate on P2. We employed the standard WALANT technique popularized by D.Lalonde:10 ml of 1 % lidocaina with 1:100,000 epinephrine (buffered at a ratio of 10 ml of lidocaine/epinephrine to 1 ml of 8.4 % sodium bicarbonate).

Post-operative management included the use of a removable thermoplastic splint to facilitate early active mobilization. A cylindrical splint was specifically designed in light flexion (20°) to mitigate stress on the volar plate and collateral ligaments, essential for reducing post-operative edema and the risk of scar adhesion and contraction. The rehabilitation regimen required the patient to remove the splint every 2–3 h during the day to perform two types of exercises aimed at managing edema and preserving the mobility of the digital chain. This approach underscores the complexities of managing significant traumatic injuries to the PIPj, necessitating both precise surgical techniques and a carefully structured rehabilitation protocol to optimize functional recovery.

## 3 Clinical findings

The clinical examination of the patient revealed significant findings related to the trauma sustained at the proximal interphalangeal joint (PIPj) of the third finger on his left hand. Upon initial assessment, the finger exhibited notable deformity and swelling. The PIPj was tender to palpation, and there was a visible and palpable dorsal dislocation of the P2 segment, confirming the diagnosis of a fracture-dislocation. Manual testing showed complete lack of active movement and elicited severe pain, indicative of the injury's impact on joint function and stability. Radiographic evaluation at the emergency department included lateral views of the injured finger, which clearly demonstrated the extent of the dislocation and the size of the volar fragment, which was about 50 % of the joint surface. These findings corresponded to a Grade 3 injury classification according to the Hasting and Carroll system. The x-rays also showed the comminution and articular involvement of the fracture, crucial for planning surgical intervention. Due to the nature of the injury and the clinical management settings, clinical photographs were taken for further assessment and to aid in surgical planning. These images were obtained with the patient's consent and under privacy regulations to ensure confidentiality and use solely for medical evaluation and treatment planning.

The surgical intervention subsequently confirmed the instability and complex nature of the fracture during the operation. A detailed intraoperative assessment revealed the challenges in achieving a stable and congruent joint reconstruction, which was addressed using adapted orthopedic hardware and meticulous surgical technique. Post-operative clinical assessments focused on evaluating the stability of the reconstruction, the alignment of the joint, the swelling, and the progression of healing. Functional assessments included monitoring the range of motion, both passive and active, which was crucial for guiding the rehabilitation process.

Overall, the physical examination and other clinical findings were consistent with a severe injury to the PIPj, requiring a comprehensive approach to management that included surgical repair, detailed imaging, and ongoing functional assessments to ensure optimal recovery and return to function. This case study adheres to the SCARE (Surgical Case Report) guidelines for reporting surgical case studies [20,21] (Table 1).

**Fig. 2:**
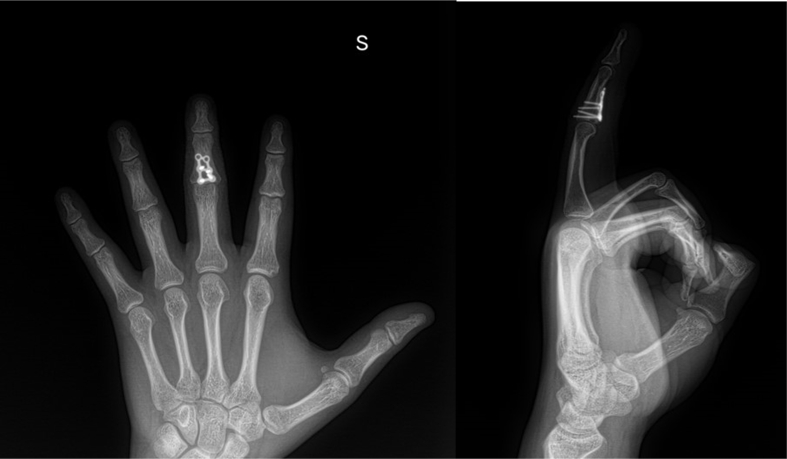
Post-operative X-ray with surgical hardware in place. A post-operative lateral X-ray of the left hand demonstrating successful placement of the adapted Medartis TriLock plate at the PIP joint of the third digit. The image shows the alignment and stabilization achieved through the surgical procedure, indicating the beginning of the patient's journey to recovery.

**Fig. 3:**
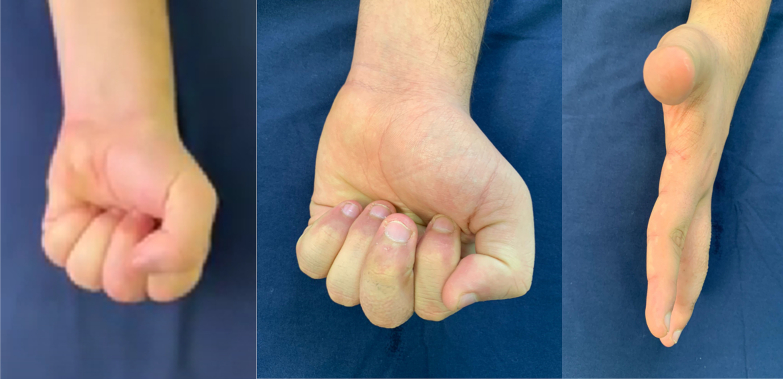
Functional recovery post PIP joint fracture-dislocation surgery. A series of clinical photographs displaying the patient's hand from different angles showcasing successful functional recovery post-surgery. From left to right: fist clenching, hand resting, and side view of the extended hand, all indicating a good range of motion and alignment after the therapeutic intervention.
Diagnostic Challenges: Rapid referral to specialized hand surgery was crucial for optimal outcomes. Financial costs related to comprehensive care could pose challenges, though no specific cultural issues were reported.
Diagnostic Reasoning: The diagnosis of fracture-dislocation was confirmed by clinical presentation and imaging, with differential diagnoses including simple dislocation and ligamentous injuries. The significant volar fragment and joint instability necessitated surgical intervention.
Prognostic Characteristics: Without surgery, the prognosis is poor due to risks of dysfunction, instability, and osteoarthritis. Timely surgical treatment aimed to restore stability and function, enhancing long-term outcomes.
Therapeutic Intervention
  Pre-intervention Considerations: The patient, a healthy young adult, required minimal pre-operative preparation and had no medication adjustments.
  Types of Interventions Deployed: *Surgical Intervention:* A Medartis TriLock plate, typically for the proximal phalanx, was adapted for the middle phalanx to stabilize the fracture and restore function. Antibiotics and analgesia were administered to prevent infection and manage pain.
  Physiotherapy: Early mobilization post-surgery was initiated to prevent stiffness and promote recovery. The rehabilitation regimen included exercises for managing edema and preserving tendon gliding. Edema management involved positioning the hand in flexion to reduce swelling and improve tissue gliding. Active mobility exercises included the Hook Fist and Full Fist exercises, proposed by Evelin Mackin, targeting differential and concentric gliding of the flexor tendons, and Selective Deep Flexor Gliding with the PIPJ slightly flexed to enhance deep flexor movement.
  Peri-intervention Considerations: *Anesthesia:* The WALANT technique allowed for immediate movement assessment and pain control. *Surgical Procedure:* The procedure involved a Bruner incision on the palmar side of the PIPJ, careful dissection, and hardware placement. Absorbable sutures secured the volar plate.
  Post-intervention Considerations: The patient was advised to keep the limb elevated, manage pain, and avoid strenuous use of the hand until cleared by the surgical team. Initial recovery was inpatient, followed by outpatient follow-ups.
  Degree of Novelty: Adapting the Medartis TriLock plate for a non-standard application demonstrated innovative surgical practice tailored to the injury's specifics.

Diagnostic Methods: *Physical Examination:* The patient's left hand showed swelling, deformity, tenderness, and a dislocated third finger at the PIP joint. The manual test indicated complete immobility and severe pain, suggesting a complex injury. *Radiological Imaging:* Initial X-rays revealed a severe PIPJ fracture-dislocation with a large volar fragment, essential for planning the surgical approach. *Postoperative Imaging:* Follow-up X-rays (Fig. 2, Fig. 3) confirmed proper hardware placement and joint alignment post-surgery.

**Table 1:** Timeline.

Date	Event
October 14, 2023	Injury occurred during a soccer game due to a violent impact with the ball.
October 14, 2023	Patient presented to the local emergency room immediately after the injury.
October 14, 2023	Initial X-rays taken; fracture-dislocation of the PIPj of the third finger diagnosed.
October 14, 2023	Plaster splint applied for immobilization; referral to hand surgery at the Hospital.
October 16, 2023	Surgical consultation at the Hospital.
October 16, 2023	Surgical intervention performed using WALANT technique and Bruner incision.
October 18, 2023	First post-operative dressing change; removable thermoplastic splint applied for early mobilization.
One week post-op	First follow-up appointment; excellent initial recovery noted in clinical assessment.
Two days later	First physical therapy session to begin structured rehabilitation exercises.
Monthly follow-ups	Ongoing monthly assessments to monitor healing, function, and range of motion.
March 20, 2024	Final reported assessment showing excellent range of motion and functional recovery.

## 4 Follow-up and outcomes

Following the surgical repair of a fracture-dislocation in the proximal interphalangeal joint (PIPj) of the third finger, the patient underwent a structured follow-up regimen to assess recovery and long-term outcomes. Clinician-assessed and patient-reported evaluations were conducted at regular intervals: weekly for the first month post-surgery, monthly up to six months, and then annually to monitor long-term recovery and function. The outcomes were primarily measured by improvements in pain levels, range of motion, joint stability, and overall hand function. Radiological assessments, performed during these follow-ups, showed proper alignment and healing of the fracture, with images from the one-year follow-up confirming the complete healing of the fracture with no signs of joint degradation.

Given the nature of the injury and treatment, ongoing annual monitoring was recommended to assess for any long-term complications such as the development of osteoarthritis, which is common in such cases. The patient's adherence to post-operative instructions, including medication, use of a splint, and engagement in physiotherapy, was excellent, as evaluated during follow-up visits. This adherence was facilitated by clear instructions and the initial use of WALANT anesthesia, which permitted early mobilization—key to the patient's quick recovery. The patient reported high satisfaction with the pain management and functional outcomes, highlighting the tolerability and effectiveness of the treatment plan.

Minor complications included transient swelling and mild pain post-operatively, managed effectively with NSAIDs and adjustments in the physiotherapy regimen. There were no severe adverse events such as infection, intense pain, or hardware failure. The complications were classified as minor (Clavien-Dindo Classification Grade I) [22].

The surgical procedure itself was efficient, lasting about 90 min with minimal blood loss and no need for re-exploration or revision surgeries. The wound healing process was smooth, with no complications, further facilitating a speedy recovery.

Overall, this case exemplifies the success of immediate and specialized surgical intervention followed by diligent post-operative care and rehabilitation. At the one-year follow-up, the patient had regained full function of the injured finger and expressed high satisfaction with both the surgical outcome and the rehabilitation process. This successful management underscores the importance of a well-coordinated approach in treating complex hand injuries, ensuring optimal functional restoration and patient satisfaction.

## 5 Discussion

In the management of proximal interphalangeal joint (PIPJ) fracture-dislocations, various surgical techniques and fixation devices are available. Traditional options include closed reduction and splinting, dynamic external fixation, and internal fixation using screws or plates specifically designed for phalangeal fractures. Plates designed for the middle phalanx do exist; however, in this case, a Medartis TriLock plate, typically used for the proximal phalanx, was adapted for the middle phalanx. This decision was based on the specific anatomical and fracture characteristics, which required a robust and stable fixation that the adapted TriLock plate could provide effectively. The choice not to use a plate designed explicitly for the middle phalanx was influenced by the need for better mechanical stability and the availability of the TriLock plate during the surgery. The use of the WALANT (wide awake local anesthesia no tourniquet) [23] technique was a significant strength in this case, facilitating immediate postoperative mobility and effective pain assessment without the risks linked to general anesthesia or tourniquet use. The Medartis TriLock plate, originally for dorsal placement on the proximal phalanx, was innovatively used on the palmar side of the middle phalanx. While early rehabilitation is possible with block anesthesia and a tourniquet, the WALANT technique allows intraoperative assessment of joint movement. This ensures optimal fixation and immediate mobilization, enhancing surgical outcomes and informing better rehabilitation protocols.

This not only contributed to rapid recovery but also enhanced patient satisfaction concerning pain management. Moreover, the innovative adaptation of a Medartis TriLock plate [24], typically utilized dorsally on the proximal phalanx, for volar placement on the middle phalanx, demonstrated a creative and effective solution for a complex fracture configuration [25,26].

However, the application of this surgical approach comes with limitations. The success of such an innovative technique relies heavily on the surgeon's expertise and familiarity with the device potentially limiting its widespread adoption in less specialized settings. Furthermore, using hardware in an off-label manner, although effective in this instance, would benefit from further validation studies to ensure safety and efficacy across a broader patient demographic.

The potential risks of applying this surgical technique to a larger population include complications like improper plate placement, increased surgical complications, or hardware failure if not executed meticulously. Longer-term complications, such as joint stiffness or osteoarthritis, are common in severe joint injuries and might be exacerbated by suboptimal surgical interventions.

Relevant literature supports the utilization of early mobilization and specific hardware for stabilizing complex PIPj fractures to optimize outcomes. However, innovative hardware uses, as seen in this case, are less commonly documented, indicating an area ripe for further research and hypothesis testing about orthopedic hardware adaptation [[27], [28], [29]].

Postoperative outcomes using the WALANT technique with the Medartis TriLock plate show improved range of motion (ROM), lower pain scores (VAS), and better functional recovery compared to traditional methods. While early rehabilitation is possible with block anesthesia and a tourniquet, WALANT allows immediate postoperative assessment and mobilization, enhancing pain management and reducing tourniquet-related discomfort. This promotes faster functional recovery and superior long-term outcomes. Long-term follow-up is crucial in assessing the durability of the surgical intervention, the potential development of osteoarthritis, and the sustained functional outcomes. Although the initial results are promising, the short follow-up does not provide sufficient data to conclusively determine the long-term prognosis. Future studies should include extended follow-up periods to better understand the implications of this innovative surgical approach over time.

The conclusions drawn from this case are based on the successful outcomes observed post-treatment, both functionally and as reported by the patient, alongside the innovative surgical technique used and supported by comprehensive literature.

The primary “take-away” lessons from this case include the effectiveness of innovative surgical techniques that require a deep understanding of hardware design limitations, the benefits of early mobilization using techniques like WALANT to enhance recovery, and the importance of a holistic care approach, incorporating specialized surgical techniques, detailed follow-up, and personalized rehabilitation for managing complex injuries effectively.

Overall, this case underscores the significance of innovation, precision, and comprehensive care in surgical practice, offering valuable insights for the treatment of similar complex hand injuries.

## 6 Patient perspective

The patient, a young soccer goalkeeper, shared a positive perspective on his treatment for a proximal interphalangeal joint (PIPj) fracture-dislocation. He was particularly appreciative of the effective pain management facilitated by the WALANT technique, which allowed for immediate pain assessment and minimal discomfort post-surgery. The early mobilization and personalized physiotherapy regimen were crucial in his rapid recovery, enabling him to regain full functionality of his finger quickly. He also expressed confidence in the surgical team, appreciating the clear communication and the innovative approach used in his treatment. Overall, the patient reported high satisfaction with the seamless integration of surgical expertise, effective pain management, and proactive rehabilitation, which collectively contributed to a successful recovery.

## Informed consent

Informed consent has been obtained from all individuals included in this study.

## Ethical approval

Our institution does not require ethical approval for reporting individual cases or case series.

## Funding

Authors state no funding involved.

## Guarantor

Roberto Tedeschi.

## CRediT authorship contribution statement

All authors have accepted responsibility for the entire content of this manuscript and approved its submission.

## Declaration of competing interest

Authors state no conflict of interest.
